# Self-Sustainable IoT-Based Remote Sensing Powered by Energy Harvesting Using Stacked Piezoelectric Transducer and Thermoelectric Generator

**DOI:** 10.3390/mi14071428

**Published:** 2023-07-15

**Authors:** Wasim Dipon, Bryan Gamboa, Maximilian Estrada, William Paul Flynn, Ruyan Guo, Amar Bhalla

**Affiliations:** Department of Electrical and Computer Engineering, University of Texas at San Antonio, San Antonio, TX 78249, USA; zzn341@my.utsa.edu (B.G.); maximilian.estrada@my.utsa.edu (M.E.); paul.flynn@my.utsa.edu (W.P.F.); ruyan.guo@utsa.edu (R.G.); amar.bhalla@utsa.edu (A.B.)

**Keywords:** remote sensing, IoT transceiver, gateway, the things network (TTN), stacked PZT transducer, thermoelectric generator (TEG), energy converter

## Abstract

We propose a self-powered remote multi-sensing system for traffic sensing which is powered by the collective energy harvested from the mechanical vibration of the road caused by the passing vehicles and from the temperature gradient between the asphalt of the road and the soil underneath. A stacked piezoelectric transducer converts mechanical vibrations into electrical energy and a thermoelectric generator harvests the thermal energy from the thermal gradient. Electrical energy signals from the stacked piezoelectric transducer and the thermoelectric generators are converted into usable DC power to recharge the battery using AC-DC and DC-DC converters working simultaneously. The multi-sensing system comprises an embedded system with a microcontroller that acquires data from the sensors and sends the sensory data to an IoT transceiver which transmits the data as RF packets to an ethernet gateway. The gateway converts the RF packets into Internet Protocol (IP) packets and sends them to a remote server. Laboratory and road-testing results showed over 98% sensory data accuracy with the system functioning solely powered by the energy harvested from the alternative energy sources. The successful maximum transmission distance obtained between the IoT, and the gateway was approximately 1 mile, which is a considerable transmission distance achieved in an urban environment. Successful operation of the self-powered multi-sensing system under both laboratory and road conditions contributes considerably to the fields of energy harvesting and self-powered remote sensing systems. The energy flow chart and efficiency for the steps in the system were found to be mechanical power from vehicles to the energy harvester of 0.25%, stacked PZT transducer efficiency was found to be 37%, and for the TEGs the efficiency is 11%. AC-to-DC and DC-to-DC converters’ efficiencies were found to be 90% and 11%. The wireless communication RF transceiver efficiency was found to be 62.5%.

## 1. Introduction

**Figure 1:**
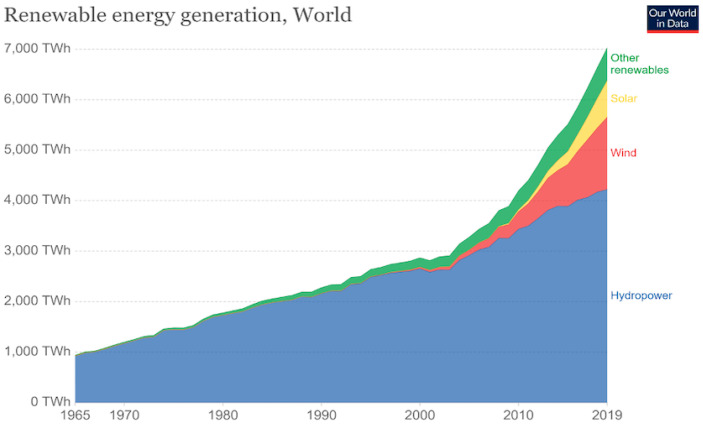
Categories and amount of renewable energy generation (2020) [1].

The advent of smart technologies in all aspects of our life has given an incredible boost to the use of remote sensing with real-time data transmission ability and high data accuracy. Remote sensing is in widespread use among various industries, research works, and in our smart homes. Sensing and data acquisition are parts of an essential revolution taking place to automate every walk of our lives. From space exploration to underwater research, every venture is assisted to a great extent by the use of sensing technologies and data acquisition. Remote sensing, in particular, is widely used across research works and industries for acquiring data wirelessly from the sensor. This not only reduces the installation cost by eliminating wires but also allows users to be stationed far from the site of sensing. Remote sensing is the backbone of IoT technology across all applications and, hence, it is an integral part of automation where IoT devices are at the forefront. Most remote sensing that is in use or in the process of being used depends on either batteries or conventional power to run them. Batteries have a lifetime and then need to be disposed of while the devices are replaced with new batteries. This action adversely affects the climate and environment by disposing of toxic materials such as lead. As the widespread use of IoT devices is taking place, more batteries will need to be disposed of, consequently contributing to dire climate and environmental scenarios. The conventional perspective of fighting this climate and environmental effects is to focus on increasing the use of already widely accepted renewable energy sources. These are mainly solar, wind, thermal, and hydropower. Ritchie, Hannah, Max Roser, and Pablo Rosado mentioned this in their paper [1]. Figure 1 shows the energy generation for each category of renewable energy sources until the year 2019 from a survey done by British Petroleum (BP). This indicates the vast difference between hydro and wind energy generation with solar and other renewable sources. The other renewables also consist of biofuel and thermal sources. However, there are many other sources of energy in the surroundings that can be harvested and used for various applications. As the data show, there is not much work done or underway to harvest and use these alternative energy sources which are readily available in the surroundings.

Lukai Guo and Qing Lu mentioned the concept of collective energy harvesting from pavements using piezoelectricity and thermoelectric generators in their review paper [2]. They stated that most studies on the piezoelectric effect application with piezoelectric transducers (PZTs) showed its limitation in the amount of instantaneous electricity output, while a limited number of studies indicated that a pipe system cooperating with a thermoelectric generator (TEG) may produce more electric power and so has more application potential in energy harvesting pavements. As per their paper, a piezo-thermoelectric (PZT-TEG) system is most effective given the cost–benefit consideration compared to only having PZTs embedded in the pavements. Lallmamode, MA Mujaahiid, and AS Mahdi Al-Obaidi presented a PZT-TEG system to harvest energy from vehicle transportation on highways [3]. However, the electrical energy output from their system, which is 0.2 mW, is not enough to sustain the functional operation of a remote sensing system. Han, Yanhui, Yue Feng, Zejie Yu, Wenzhong Lou, and Huicong Liu also presented a piezoelectric energy harvesting sensor network deployed in a weak vibration environment [4]. The system they presented is very complex with a battery-powered voltage controller. Also, the energy conversion efficiency is reported to be 42%, which is not efficient enough considering the energy conservation requirement for any alternative energy harvesting system. Mysorewala, Muhammad Faizan, Lahouari Cheded, and Abdul Rahman Aliyu also mentioned exploiting hybrid energy harvesting techniques (for example, using wind–solar–thermal energies for outdoor harvesting and radiation vibration for indoor harvesting, with both sets of combined energy sources working either in conjunction with or independently of the normally used batteries) as these combined techniques tend to compensate for one another, thus increasing the likelihood of securing an uninterruptible energy source for the wireless sensor network [5]. Shaikh, Faisal Karim, and Sherali Zeadally, in their comprehensive study of different energy harvester systems for wireless networks, mentioned electrostatic or piezoelectric generators as harvesters for harvesting mechanical vibrations [6]. The piezoelectric generator they stated has an output of 115.2 mW. This generated power is insufficient to provide sustainable energy to a relatively long-range wireless communication remote sensor since communication consumes the most energy of a remote sensing system. This paper presents research that focuses on harvesting mechanical vibrations from roadways and the thermal gradient between the asphalt on the road and the soil underneath. The energies harvested from these sources are then converted into electrical power to run an IoT-based remote multi-sensing system. Transducers are required to convert the mechanical vibrations and thermal gradient into electrical power. In this research, we are using a stacked PZT (lead zirconate titanate) transducer and a combination of thermoelectric generators (TEGs) to convert the mechanical vibrations and thermal gradient, respectively, into electrical energy. An earlier work of the authors used the same stacked PZT transducer to conduct multi-sensing, however, using Bluetooth low energy (BLE) as the communication protocol [7]. Thus, the communication range was limited to approximately 10 feet. This research aims to overcome and resolve some of the shortcomings of contemporary remote sensing systems that are using alternative energy source harvesting to power relatively long-range wireless communication protocols for remote sensing. Improvements in the areas of material engineering of the PZT transducer to the heat sink design are incorporated to produce sufficient energy that can sustain a functional IoT-based remote multi-sensing system.

## 2. Materials and Methods

### 2.1. Energy Harvesters: Stacked PZT Transducer and Thermoelectric Generators (TEGs)

The generation of electrical charges in response to applied mechanical stress is known as the direct piezoelectric effect, while the generation of mechanical strain in response to electrical charges is known as the converse piezoelectric effect [8,9]. The electromechanics underlying the direct piezoelectric and converse piezoelectric effects can be best explained with Equations (1) and (2), where x is the mechanical strain [m/m], E is the electric field [V/m], and, in Equation (2), P represents electric displacement [C/m^2^] and X is the stress [N/m^2^]. In both equations, d is the piezoelectric coefficient [C/N or m/V] which determines the sensitivity of the response of the sample under stress or in an applied electric field [8]. These relationships of piezoelectric materials can be utilized to make piezoelectric materials function as energy harvesters.

(1) $${Direct\;piezoelectric\;effect:P}_{i}=d_{kij}X_{jk}\;$$

(2) $${Converse\;Piezoelectric\;effect:x}_{ij}=d_{ijk}E_{k}$$

**Figure 2:**
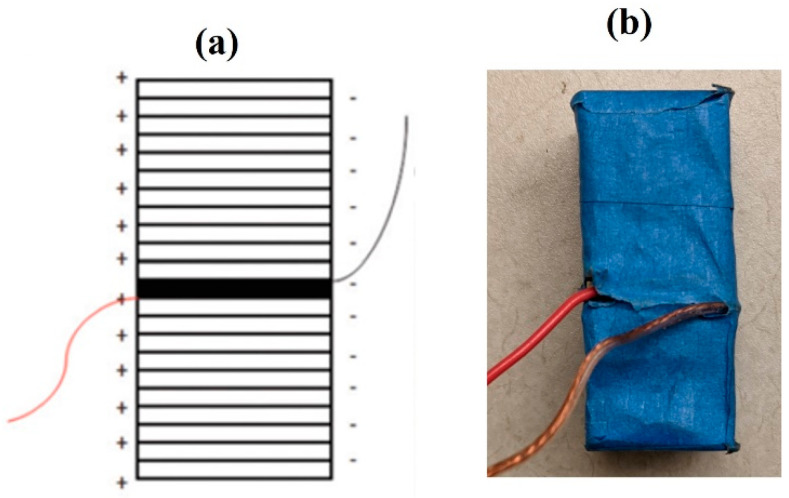
(**a**) Block diagram of the assembled stack, (**b**) final stacked PZT transducer [7].

The stacked PZT transducer is custom fabricated from thin plates of PZT-5H-based soft PZT materials. The advantage of using PZT-5H is that it is easily polarized compared to PZT-8, which is a hard PZT material. Figure 2a shows the design of the assembled stacked PZT transducer. The transducer consisted of 21 active plates and one insulating plate. Each PZT plate was of a length and width of 20 mm and a thickness of 2 mm. To ease the alignment issues during fabrication, the stacks were first built in separate 10 and 11 plates and then combined. Indium was chosen as the electrode bonding between the plates and is connected in a way that forms parallel connections between the plates. The final fabricated stacked PZT transducer is shown in Figure 2b, with the dimensions h = 50 mm and l = w = 20 mm [8].

Gamboa et al. fabricated a stacked PZT transducer for optimized energy harvesting [8] and compared it with various piezoelectric transducers for optimized energy harvesting. Gamboa et al. conducted testing and evaluation of three types of stacked PZT transducers: a 1:3 composite stacked PZT transducer, a specially designed and fabricated stacked PZT transducer, and a commercially available stacked sample. PZT-5H samples were used to assemble the stacked PZT transducer. Piezoelectric material PZT-5H is used to harvest energy in wireless sensor networks due to its very high permittivity and sensitivity properties. These materials have electromechanical properties which are used to harvest energy [9]. The fabricated transducer gave maximum power density per unit of transducer volume, measured at 0.615 mW/mm^3^ at 965 KN/m^2^ (140 psi). Considering power density per unit of transducer volume as the more appropriate way of determining the type of stacked transducer to be used, the fabricated stacked PZT transducer was chosen to be used for this research. The PZT plates are mechanically in series and electrically in parallel to increase the current from the stacked PZT transducer.

**Figure 3:**
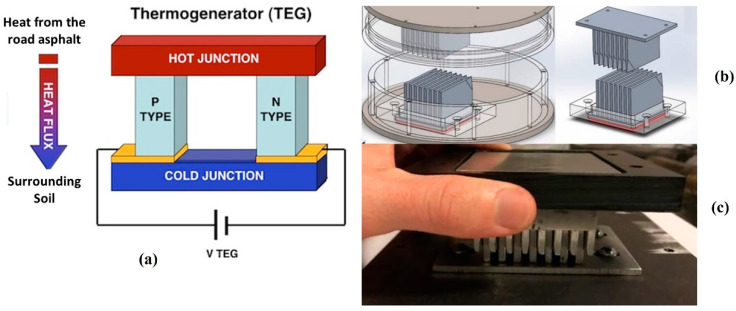
(**a**) Illustration of the Seebeck effect. (**b**) COMSOL model of the heat sink in the module along with heat sink isolated. (**c**) The fabricated TEG and heat sink module [10].

Thermoelectric generators (TEG) are solid-state semiconductor devices that convert a temperature difference and heat flow into a useful DC power source. Thermoelectric generator semiconductor devices utilize the Seebeck effect to generate voltage. This generated voltage drives electrical current and produces useful power at a load. The Seebeck effect is the generation of electricity between a thermocouple when the ends are subjected to the temperature difference between them. A combination of four thermoelectric generators is used in the research. Bismuth telluride (Bi_2_Te_3_) TEGs were selected due to the high figure of merit. The figure of merit is the number used to determine the efficiency of a TEG to convert a temperature difference into useful electrical energy. The figure of merit for different materials varies with temperature. Considering the system will be deployed in Texas, where maximum temperature difference occurs during summertime causing the asphalt temperature to reach 400 K, bismuth telluride TEGs have the highest figure of merit at this temperature. Figure 3a illustrates the Seebeck effect considering the temperature gradient between the asphalt and the surrounding soil. The heat flows from the asphalt to the soil underneath. Heat sinks are used between the top and bottom plates to carry the heat from the asphalt to the top metal junction of the TEGs. The bottom metal junction of the TEGs is at the same temperature as the soil underneath the asphalt. Figure 3b shows the COMSOL model of the heat sink concept as a module with the TEGs as well as isolated. Figure 3c shows the fabricated heat sink module along with TEGs inside. The heatsink design was validated by COMSOL simulation, and the model indicated the TEG was able to increase the temperature difference across the TEG from 0.1 °C to 1.1 °C [10]. The TEGs were tested with the module in direct sunlight. A total of 4 TEGs are divided into 2 groups, each comprising 2 TEGs connected in parallel are in series with the other group, making 4 TEGs in total. This combination is done to attain optimized voltage and current from the TEGs. The stacked PZT transducer has an energy conversion efficiency of 37% and each TEG has an efficiency of 11%.

### 2.2. Converters for Stacked PZT Transducer and TEGs

**Figure 4:**
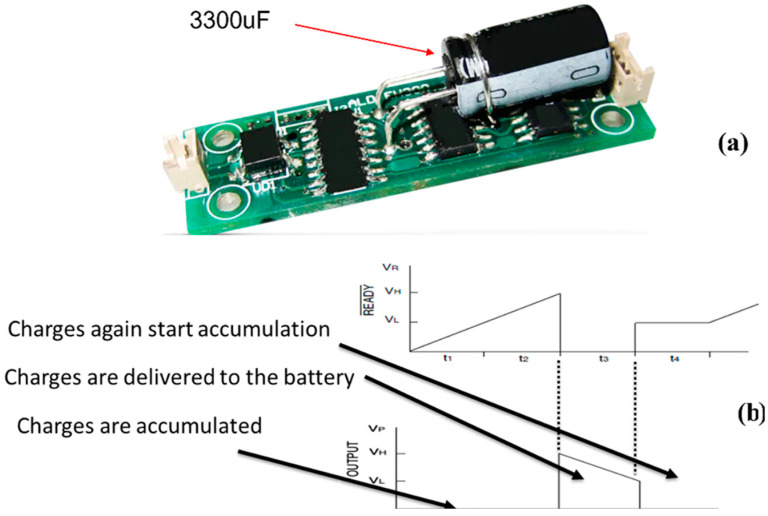
(**a**) EH301A energy harvester with 3300 uF storage capacitor. (**b**) Charging and discharging cycle of the EH301A energy harvester.

The electrical signals from the stacked PZT transducer and the TEGs are sinusoidal and DC, respectively. Hence, it is required to have a combination of AC-to-DC and DC-to-DC boost conversions in the system. The voltage from the stacked PZT transducer is very high and, hence, needs to be reduced. The voltage from the TEGs is low and, hence, is required to be boosted to the charging voltage of the battery. The AC-to-DC conversion aims at collecting as much charge as possible from the PZT. As per the maximum power transfer theorem, maximum power transfer from the source to the load is attained when the impedances of the source and the load match. The stacked PZT transducer can be represented as an electrical circuit consisting of a current source, a resistance, and a capacitor all in parallel. The electrically parallel combination of the PZT plates in the stacked PZT transducer creates an increase in the capacitive impedance of the circuit which leads to lower total impedance of the source. This is an important feature as it facilitates impedance matching with the impedance of the AC-to-DC converter, which is typically lower. Consequently, higher charge collection is made possible due to the impedance matching. The AC-to-DC converter also abruptly and efficiently reduces the high voltage generated by the stacked PZT transducer by rapidly collecting the charges once they are generated. Since the transducer acts as a capacitor, its voltage is a function of the charges accumulated in the transducer. Once these charges are extracted from the transducer to charge the battery, the high voltage generated by the transducer drops abruptly. Hence, a capacitor-based converter is selected since a capacitor acts as a short circuit at the start. Consequently, the generated charges are collected immediately to drop the voltage of the transducer. An EH301A (Sourced from Advanced Linear Devices Inc., Sunnyvale, CA, USA) energy harvester, shown in Figure 4a, is used as the AC-to-DC converter for the stacked PZT transducer. It is equipped with a 3300 μF storage capacitor which is charged by the charges from the stacked PZT transducer. The output voltage rises until it reaches the charging voltage of the battery when the accumulated charges are transferred into the battery until the output voltage drops below the charging voltage and the cycle repeats. This cycle and different stages are shown in Figure 4b, where V_P_ represents the output voltage of the converter which switches between V_H_ and V_L_ representing high and low voltage levels. The conversion efficiency of the AC-to-DC converter was found to be 90%.

**Figure 5:**
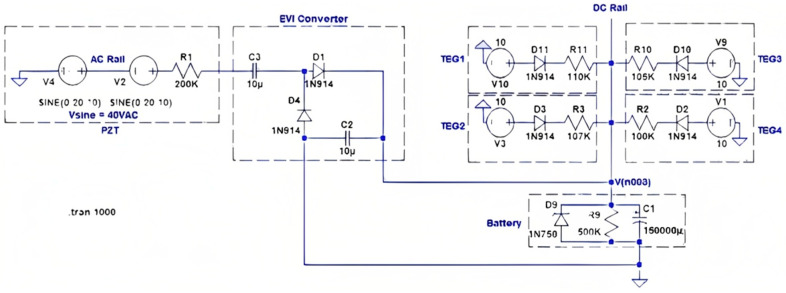
Pspice model design of an equivalent circuit of the system for verification.

**Figure 6:**
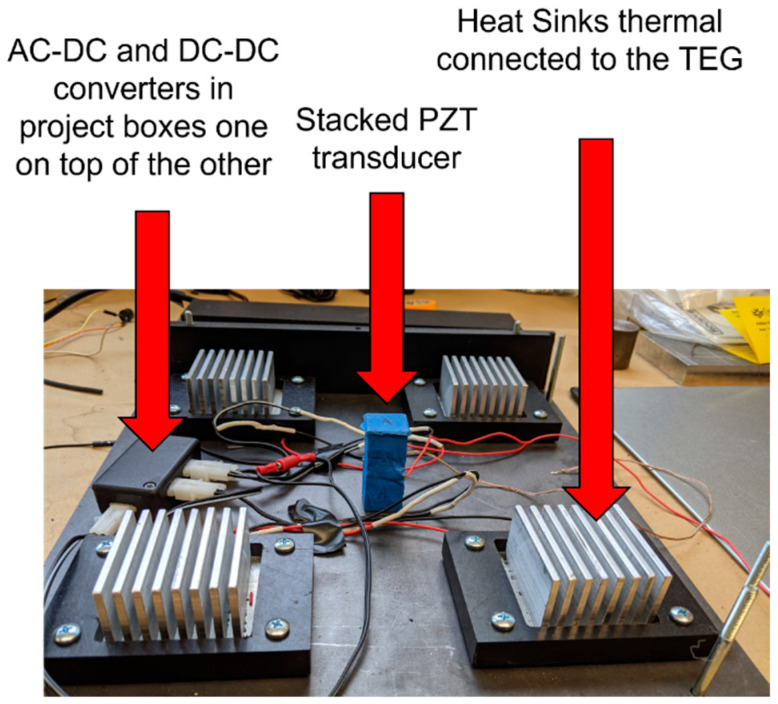
Hybrid Integrated Sensing and Energy Conversion (HiSEC) module with the stacked PZT transducer, heat sinks for the TEGs, and the converters.

The voltage from the TEGs, which is 120 mV, is very low and needs to be boosted to the charging level of the battery. ELC-BVB120 boost convert is selected for the TEGs because its conversion efficiency was experimentally found to be 86% while converting 120 mV to about 4 V with a load. The objective was to have both AC-to-DC and DC-to-DC boost converters operate simultaneously, charging the rechargeable battery pack of NiCad batteries. The Pspice model for the combined converter design is shown in Figure 5. It was recorded that multi-sourcing energy is very possible by the carefully designed electronic module. Minimum energy sourced from harvesters that can be converted to collect energy cumulatively to charge a battery thus also proved to have high efficiency of the module. Four TEGs are connected to the DC-rail using the concept that was presented before. Current responses are equivalent to what one should expect from a TEG at the 10 V (open circuit) output of the boost converter. A low thermal variant, that is, a small temperature difference across the TEG plates, is considered in this simulation because the soil is an excellent thermal conductor and, hence, the temperature difference is assumed to be small between the asphalt on the road and the soil underneath. To make more rigorous verification of the design, TEGs were given some uneven internal impedance to create non-uniform current responses. All resistors are used in line with the energy source of devices to control current and make them equivalent to the actual test results obtained from the boost converters. Since boost converters were not incorporated into the schematic, equivalent circuits were designed in the schematic. All multi-sources at PZTs’ AC-rail and TEGs’ DC-rail are acting to charge the battery flawlessly. Continuously feeding current is possible by this technique, because a rechargeable battery is a very large capacitor in real life. The real-time tested power was observed to be more than expected, with an improvement of 30.27% in efficiency. Inductive and capacitive converter combinations might have produced better impedance responses, resulting in a higher current to charge the battery. Figure 6 shows the stacked PZT transducer, TEG heatsink modules, and the converters inside the mechanical enclosure called HiSEC (Hybrid Integrated Sensing and Energy Conversion) module.

### 2.3. Sensor Hardware, Algorithm, and Wireless Communication

**Figure 7:**
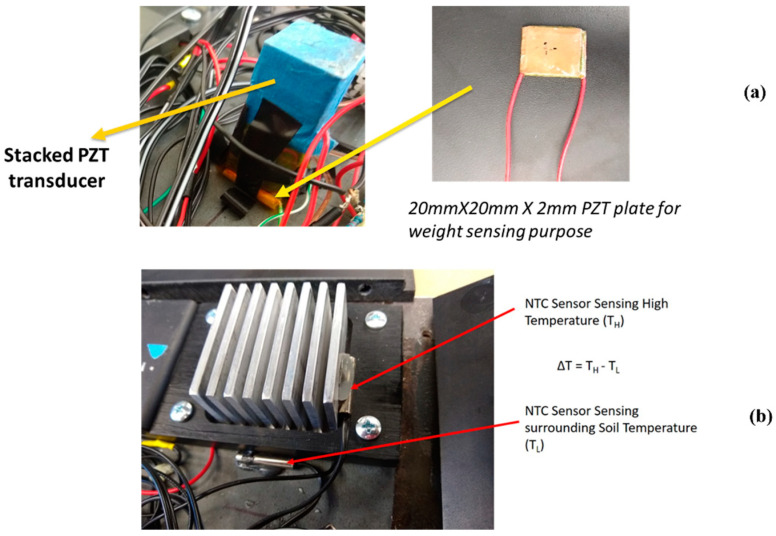
(**a**) Piezoelectric transducer-based tire pressure sensing. (**b**) Temperature difference measurement using NTC temperature sensors.

Three sensing parameters are transmitted over wireless communication. The system is developed to be deployed buried under the roadway as it is intended to harvest energy from the vehicles passing over the road and the thermal gradient between the asphalt and the soil underneath. A PZT-5H based 2 mm × 2 mm × 2 mm piezoelectric plate was used as the piezoelectric-based pressure sensor to estimate the weight of the vehicle. The PZT plate also acts as the sensor counting the axle counts of the vehicles. Two negative temperature coefficient (NTC) temperature sensors were used to read the temperature difference between the asphalt and the soil. Figure 7a shows the piezoelectric pressure sensor and Figure 7b shows the NTC based temperature sensors installed on the heat sink and the lower plate of HiSEC. A microcontroller with an algorithm coded does all the computation required to estimate the sensing parameter values. The microcontroller used in the research is a 16 MHz ATmega328P-based microcontroller called Arduino Nano (Sourced from Microchip Technology Inc., Chandler, AZ, USA) which uses RISC architecture with an ADC resolution of 10 bits. It has a flash memory of 32 KB, of which 2 KB is occupied by the bootloader. The other smaller option was Arduino Pro Mini, which also has lower power consumption. However, Arduino Pro Mini does not come with a USB port and, hence, it is difficult to load programs onto it. Also, Arduino Nano does not occupy much space and the slightly higher power consumption compared to Arduino Pro Mini is compensated for by the ease of programming and reprogramming if required.

**Figure 8:**
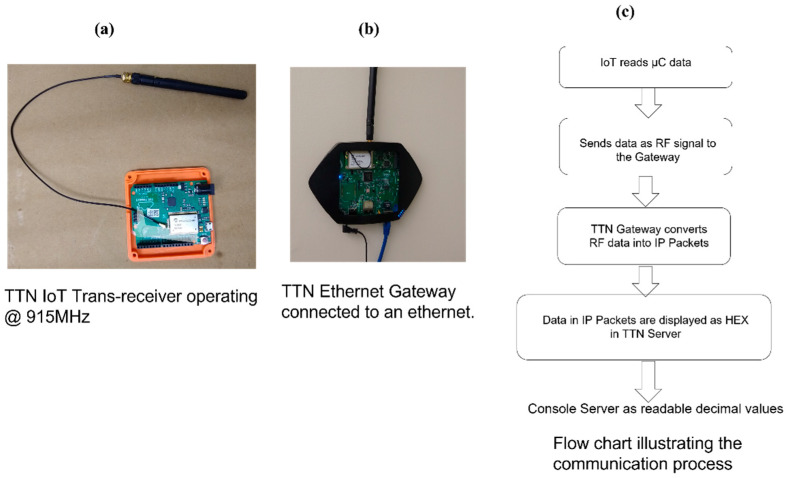
(**a**) TTN IoT transceiver. (**b**) TTN ethernet gateway. (**c**) Flow chart illustrating the communication steps.

**Figure 9:**
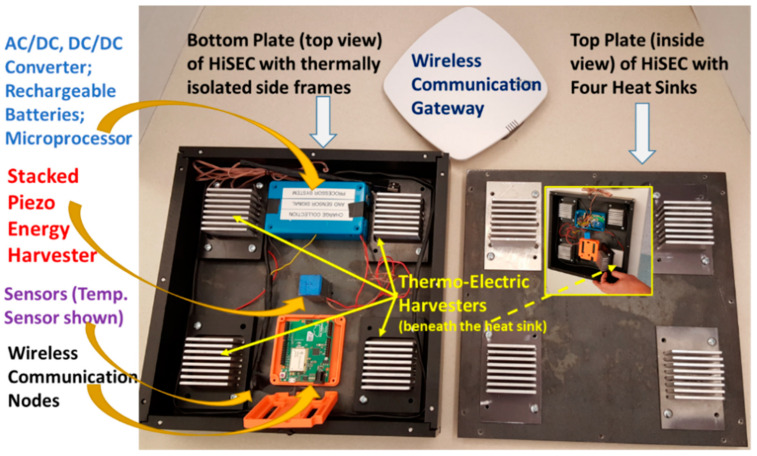
A prototype demonstration unit of HiSEC with main components labeled.

The communication protocol used in the research is LoRa WAN with an operating frequency of 915 MHz and the default server used to collect and display the sensor data is called The Things Stack or TTN server, which is a LoRa WAN network server. The LoRa WAN protocol uses Chirp Spread Spectrum (CSS) modulation technology. The hardware of the LoRa WAN consists of an IoT transceiver with a patch antenna fabricated on it which can be extended into a dipole antenna by soldering it. An ethernet gateway is connected to the internet through an ethernet connection. The IoT transceiver receives serial data from the microcontroller of the embedded multi-sensing system using a Serial Peripheral Interface (SPI) protocol embedded in the codes. The serial data are then converted into radio frequency (RF) packets and sent wirelessly to the nearby The Things Network gateway which converts these RF packets into Internet Protocol (IP) packets and transmits them to The Things Network (TTN) server as a stream of hexadecimal values. Since the hexadecimal version of the data—which is the default data type on the TTN server—is not user-friendly, a console server is established to display the sensory data in a user-friendly manner. This was done by a Java script and a free server account. This script takes the hexadecimal data from the TTN server and segregates them under the corresponding sensor category and displays them as decimal values on the console server. The hardware of the LoRa WAN and the entire communication steps are shown in Figure 8. The completed prototype of the HiSEC is shown in Figure 9.

## 3. Results and Discussion

**Figure 10:**
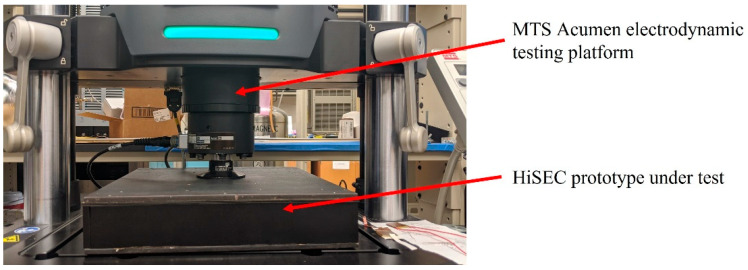
HiSEC prototype under test using MTS Acumen electrodynamic testing platform.

**Figure 11:**
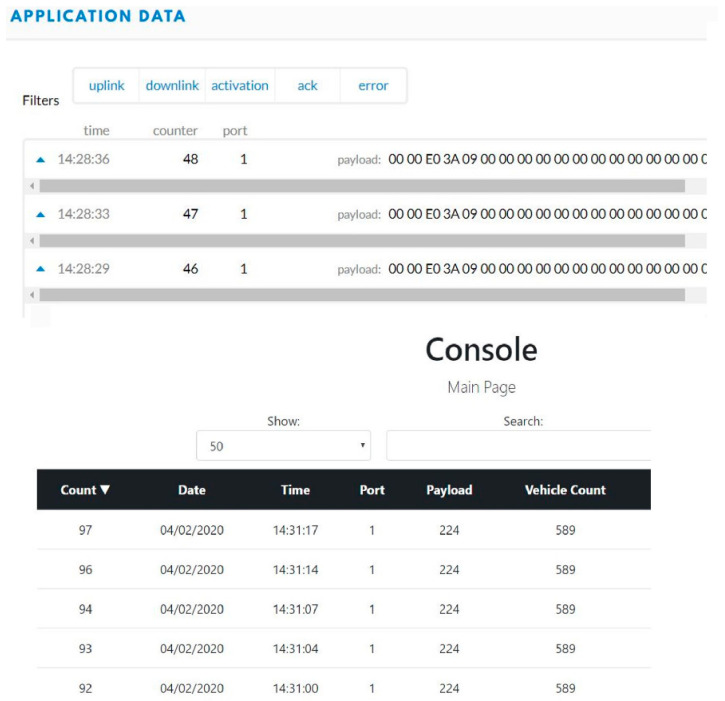
Sensory data (1.5 KN, 10 Hz, for 60 s) displayed in hexadecimal format on the TTN server and converted into a decimal format to be displayed on the console server by the Java script.

**Figure 12:**
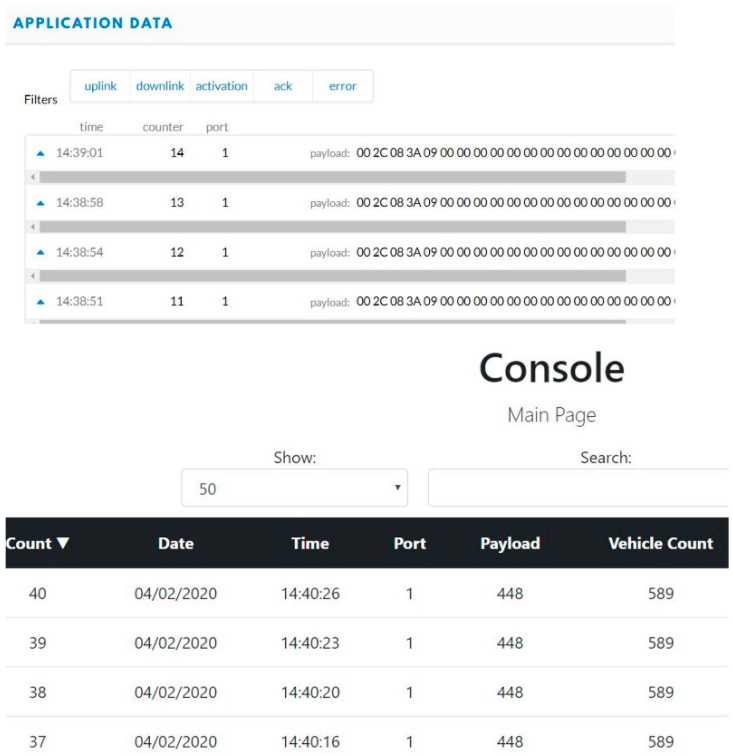
Sensory data (2 KN, 10 Hz, for 60 s) displayed in hexadecimal format on the TTN server and converted into a decimal format to be displayed on the console server by the Java script.

An MTS Acumen electrodynamic testing platform is used to test the system in a laboratory environment under various forces and frequencies (Figure 10). These forces are used to test the electrical power conversion from mechanical vibrations of the stacked PZT transducer. Real-time sensory data in hexadecimal forms and decimal forms were displayed on the TTN server and the console server, respectively. Power measurements from the stacked PZT transducer and the TEGs were also recorded under various forces and temperature differences. Several forces at different frequencies are used to test the HiSEC; also, temperatures were varied over a range to replicate the temperature gradient expected under roads in Texas. Two sensory parameters were recorded during the tests to prove the concept, vehicle weight, and axle count (represented as vehicle count). Figure 11 shows the real-time sensory data displayed on the TTN server and the console server when the MST Acumen ran with 1.5 KN of force at 10 Hz for 60 s. Similar data for 2 KN of force at 10 Hz for 60 s are shown in Figure 12. These equal 101.6 kg (224 lbs.) and 203.2 kg (448 lbs.) of forces at 10 Hz for 60 s, respectively.

**Figure 13:**
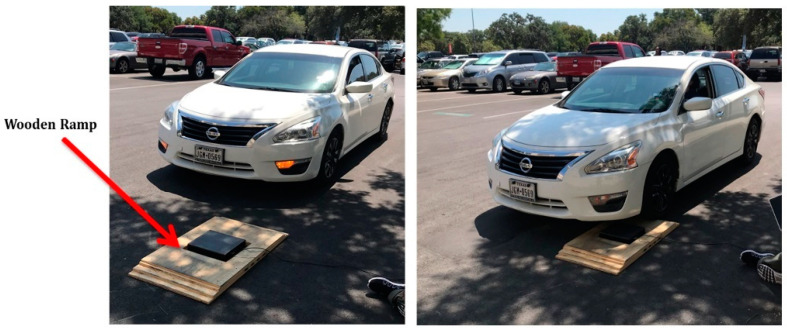
Road testing of the HiSEC module with the TTN ethernet gateway placed 30 m from the ramp.

Similar testing of the system was also conducted on the roadways with successful results. A wooden ramp was used to drive cars over it while the HiSEC was in place on the ramp, as shown in Figure 13. The maximum communication range is over a distance of approximately 1.12 km in an urban environment. However, the range was expected (more than 1 mile) to be farther than achieved without an intermittent drop in the communication channel operating at 915 MHz. Path loss will represent the amount of energy lost in free space over a distance between the transmitter and the receiver. The farther away Tx is from Rx, the lower the energy is. Path loss is usually expressed as shown in Equation (2):

(3) $$FSPL=\left(\frac{4\pi d}{\lambda }\right)\times 2=\left(\frac{4\pi df}{c}\right)\times 2$$

where FSPL = Free Space Path Loss, d = distance between Tx and Rx in meters, and f = frequency in Hertz. There is also a widely used logarithmic formula for free space attenuation as shown in Equation (3):

(4) $$FSPL\left(dB\right)=20\;\log_{10}d+20\;\log_{10}f-147.55\;\;$$

The transmitter and the receiver are located 1.12 Km, which is 1120 m when the system was tested outside. Putting d = 1120 m in the above formula, the FSPL comes out to be 27.34 dB.

**Figure 14:**
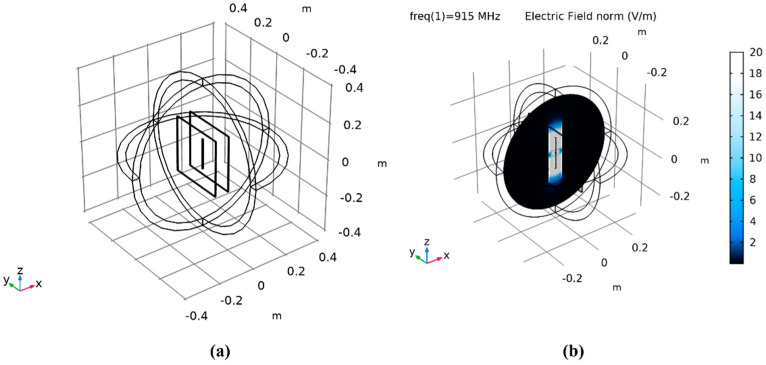
(**a**) COMSOL model of a dipole antenna between two metal plates. (**b**) COMSOL model simulation showing electric fields developing at the plates’ surfaces due to eddy currents.

COMSOL modeling and simulation of the dipole antenna between two metal plates were done to find the reasons for the shorter communication range and unstainable communication. It was concluded that the electromagnetic waves from the antenna cause eddy currents to develop at the metal plates, which inhibit the transmission of the radio frequency signals to the ethernet gateway. The COMSOL simulation result is shown in Figure 14.

The sensory data attained 98% in data accuracy for dynamic sensing of vehicle weights and axle counts, which is significantly higher than contemporary systems and conventional weight-in-motion (WIM) systems. Experimentally, the power consumption of the IoT transceiver was found to be 40 mW for 15 s each time it transmits data. That is, 0.6 W power is required for every transmission. The batteries used in the system have 800 mA-h at 3.6 V, equal to 2880 mW per hour, which is sufficient enough to restore operation during a lack of energy from the transducer. For estimating the energy that will be generated by the stacked PZT transducer and the TEGs, we considered the US-59 highway as an example. Table 1 provides the traffic data of highway US-59 by the Texas Department of Transport (TxDOT). Here, ADT is average daily traffic and ADDT is average daily truck traffic. It also gives the average truck speed, which is 29.73 ms^−1^. The stacked PZT transducer generated on average approximately 0.8 mW of power at 140 PSI. From the traffic data and the experimentally found power generation data, it can be stated that the stacked PZT transducer will generate approximately 4 W of power per day while the TEGs will generate on average 1 W of power per day. Thus, approximately eight successful transmissions can be done using the energy generated collectively from the stacked PZT transducer and the TEGs. This consumption can be reduced by increasing the period between each wireless transmission. Hence, it can be estimated that the power generation will be sufficient enough to replenish the battery power consumed by the IoT transceiver.

From the electromechanical testing of the HiSEC, it was found that the mechanical energy input from the crosshead of the testing platform to the HiSEC was 3.15 W and the 8mW energy eventually reached the stacked PZT transducer, resulting in 0.25% energy conversion efficiency. This is aligned with our estimate of the roadway scenario since it is estimated that only a small amount of the mechanical energy will reach through the asphalt, grave, soil, and then to the stacked PZT transducer. The LoRa WAN IoT transceiver output power is limited to 25 mW, resulting in an electrical-power-to-signal-power conversion efficiency of 62.5%. This research was intended to prove the concept and efficacy of an IoT-based multi-sensing system, and the efficiency of the developed system is measured in terms of the successful functionality of the system powered exclusively by the energy harvested from the stacked PZT transducer and the thermoelectric generators. The experimental results show that the system is capable of operating successfully with the harvested energy by the energy harvesters. The HiSEC module, which is an integrated system housing the energy harvesters, converters, sensors, and embedded data acquisition circuit, and the IoT transceiver is 12-inch X 12-inch X 2-inch in dimension. However, the module can be redesigned with smaller dimensions for formfitting any particular application. The application of this system can be in the fields of industries to research works where low-power remote sensing plays an essential role and where there is scarcity and inconvenience to the access of conventional power. This system can also play a part in increasing the life span of the batteries of IoT devices and, hence, will positively impact the environment. The system and the processes utilized in the HiSEC can be divided into energy domains, and each domain has its energy conversion efficiency.

## 4. Conclusions

The work presented in this paper aimed to resolve some of the most critical difficulties involving the widespread use of alternative energy sources as power sources for wireless communications in remote sensing. The HiSEC module is powered by a stacked PZT transducer acting as a mechanical energy harvester and TEGs harvesting thermal gradient. High accuracy of the sensory data was achieved for road traffic axle count, vehicle weight, and temperature difference sense. The system resolves the issues mentioned in contemporary literature by not only achieving high sensing accuracy but also ensuring sustainability in remote sensing data transmission. In comparison to the harvesters for wireless networks mentioned in the literature, which generate power in the microwatt range, the harvester in this research generates power in the range which is sufficient enough to sustain wireless communication for remote sensing. Hence, the power generated is also a significant improvement from contemporary harvesters. However, the standout factor for the developed system in comparison to the state-of-the-art is the energy-efficient way of utilizing the generated power to successfully conduct multi-sensing and wireless communication. The entire system is programmable and additional sensing capabilities can be incorporated with ease. This facilitates the adaptability of the system for multiple applications. The proof of work of this research can be utilized in many ways for other sensing applications powered by alternative energy sources. Through this research and its impact, we envisage an entirely new generation of IoT-based remote sensing based on the concept of being powered by the energy harvested from various alternative energy sources, leading to a positive impact on the environment and significantly extending battery life span. Future improvements include resolving the communication issue with an antenna between the metal plates of the HiSEC and also improving the electrical power output from the harvesters.

## Figures and Tables

**Table 1:** US-59 traffic data collected (TxDOT).

#	Parameter	Value
1	Speed Limit (mph)	75
2	Average truck speed (mph)	66.5
3	Design lane (outside lane) ADT	4208
4	Design lane (outside lane) ADTT	1599
5	Tire pressure (PSI) (vary from truck to car)	30–120

## Data Availability

All data that are relevant to this paper are provided as images.
